# Metastatic large cell carcinoma of the lung: A rare cause of acute small bowel obstruction

**DOI:** 10.1111/1759-7714.13656

**Published:** 2020-09-11

**Authors:** Min Cheol Kim, Min Hye Jang, June Hong Ahn

**Affiliations:** ^1^ Division of Gastroenterology and Hepatology, Department of Internal Medicine Yeungnam University College of Medicine Daegu South Korea; ^2^ Department of Pathology Yeungnam University College of Medicine Daegu South Korea; ^3^ Division of Pulmonology and Allergy, Department of Internal Medicine College of Medicine, Yeungnam University and Regional Center for Respiratory Diseases, Yeungnam University Medical Center Daegu South Korea

**Keywords:** Large cell carcinoma, lung cancer, metastasis, small bowel obstruction

## Abstract

Here, we report a case of acute intestinal obstruction as the initial presentation of primary lung cancer in a male patient. Abdominal computed tomography (CT) showed multiple polypoid masses and regional lymphadenopathy with small bowel obstruction. The patient underwent emergency surgery for multiple luminal malignancy with mesenteric masses. According to the various clinicopathological features, the tumor was confirmed to be metastatic large cell carcinoma originating from the lung. Large masses in the left lower lobe of the lung were identified on the chest CT after emergency surgery, and non‐small cell lung cancer (NSCLC), not otherwise specified (NOS), was finally diagnosed on biopsy through bronchoscopy.

## Graphical abstract

We report a case of acute intestinal obstruction as the initial presentation of primary lung cancer in a male patient. The patient underwent emergency surgery for multiple luminal malignancy with mesenteric masses, and immunohistochemical staining of the specimen revealed metastatic large cell carcinoma originating from the lung.

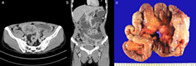

## Introduction

Lung cancer is the most commonly diagnosed cancer and the leading cause of death due to cancer.^1^ Almost half of patients with primary lung cancer are diagnosed at an advanced stage of disease.^2^ Metastasis of primary lung cancer to the gastrointestinal tract is rare, and the reported rate is 0.2%–1.7%.^3^, ^4^, ^5^ The small bowel is the most common site of metastasis in the gastrointestinal tract.^6^, ^7^ Most patients with small bowel metastasis of lung cancer have no specific symptoms, and the diagnosis is often delayed, thereby possibly leading to serious complications such as obstruction, perforation, and bleeding.^8^, ^9^ Here, we report an unusual case of metastatic large cell carcinoma of the lung in a patient who presented with acute small bowel obstruction.

## Case report

**Figure 1:**
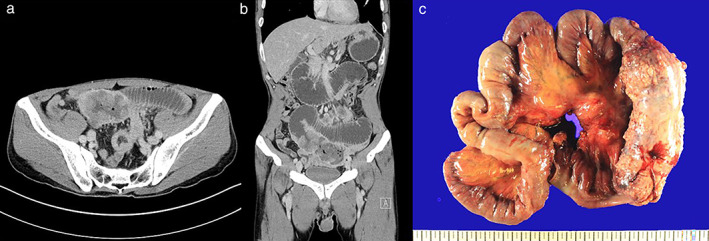
Abdominal computed tomography (CT) scan revealed multiple polypoid obstructive masses in the small bowel (approximately 15 cm in length) with a dilated proximal part of the small bowel from the tumor. (**a**) Axial view. (**b**) Coronal view. (**c**) A surgical specimen obtained after small bowel resection showed multiple obstructive masses approximately 50 cm in length with multiple mesenteric masses.

A 41‐year‐old man was admitted to the gastroenterology department with a history of repeated abdominal pain and vomiting for two weeks. He had no underlying disease but had smoked 0.5 packs a day for 20 years. Abdominal computed tomography (CT) revealed 15 cm long multiple polypoid masses in the small bowel as well as dilation of the proximal part of the mass (Fig 1a,b) During emergency surgery, multiple small bowel luminal obstructive masses, 50 cm long, were identified 30 cm away from the distal end of the Treitz ligament, and the small bowel was resected (Fig 1c).

**Figure 2:**
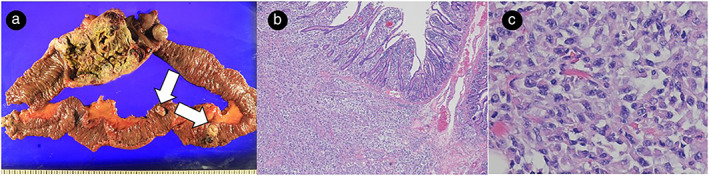
(**a**) Macroscopic image of small bowel resection (arrows indicate Several small polypoid tumors). (**b**,**c**) Microscopic images of small bowel resection. (**b**) Hematoxylin‐eosin staining (magnification × 40). (**c**) Hematoxylin‐eosin staining (magnification × 200) showed large, undifferentiated carcinoma cells with rhabdoid cytological features.

Grossly, the largest mass extensively invaded the entire layer from serosa to mucosa, and several small polypoid tumors were also identified along with the largest main mass (Fig 2a) Microscopically, all the tumors were composed of relatively homogenous large epithelioid cells with centrally located round vesicular nuclei, although some showed rhabdoid features.(Fig 2b,c).

Due to the rarity of the primary intestinal malignancy, metastatic tumor was considered to be the main diagnosis. However, primary small intestinal carcinoma, sarcoma, such as leiomyosarcoma, synovial sarcoma and gastrointestinal stromal tumor, and malignant melanoma, were also considered as differential diagnoses. Immunohistochemical staining was performed which revealed that the tumor cells were positive for cytokeratin and negative for TTF‐1, p40, chromogranin, synaptophysin, CD56, myogenin, desmin, C‐kit, HMB45 and TLE‐1. Based on these results, we concluded that the tumor was poorly differentiated carcinoma with large cell and rhabdoid features.

**Figure 3:**
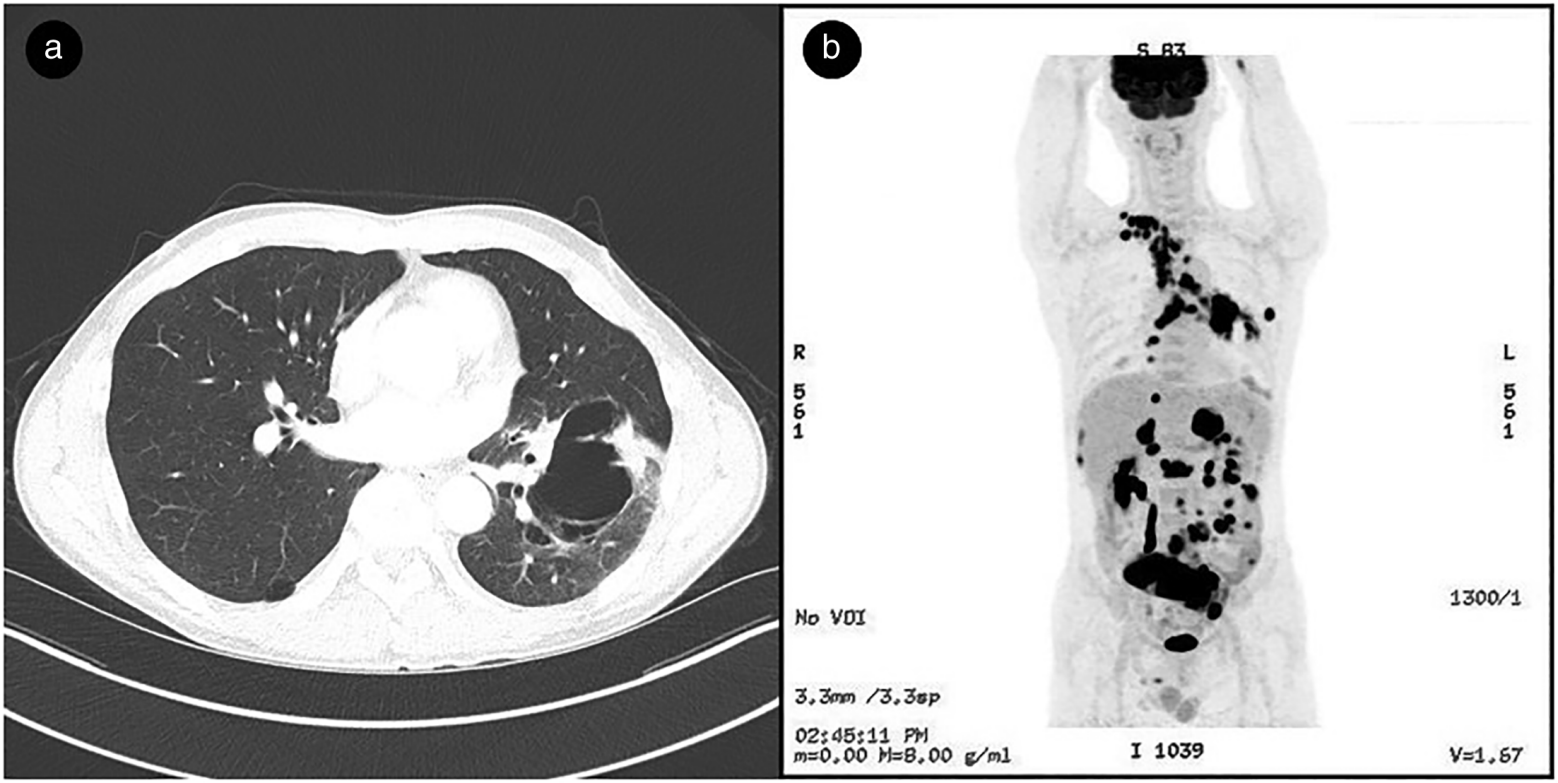
(**a**) Chest computed tomography revealed a cavitary mass 8.7 cm in size in the superior segment of the left lower lobe and multiple mediastinal lymphadenopathy. (**b**) Positron emission tomography‐computed tomography (PET‐CT) revealed a typical pattern of metastasis for non‐small cell lung cancer (NSCLC).

Chest CT showed a cavitary mass measuring 8.7 cm in the superior segment of the left lower lobe with multiple mediastinal lymphadenopathy (Fig 3a). Positron emission tomography‐CT (PET‐CT) revealed intense FDG uptake at the lung mass in the left lower lobe, multiple mediastinal lymph nodes, right pleura, small bowel, both adrenal glands, and second lumbar vertebra, which was thought to be a typical pattern of metastasis for non‐small cell lung cancer (NSCLC) (Fig 3b). Brain magnetic resonance imaging revealed no evidence of metastasis.

**Figure 4:**
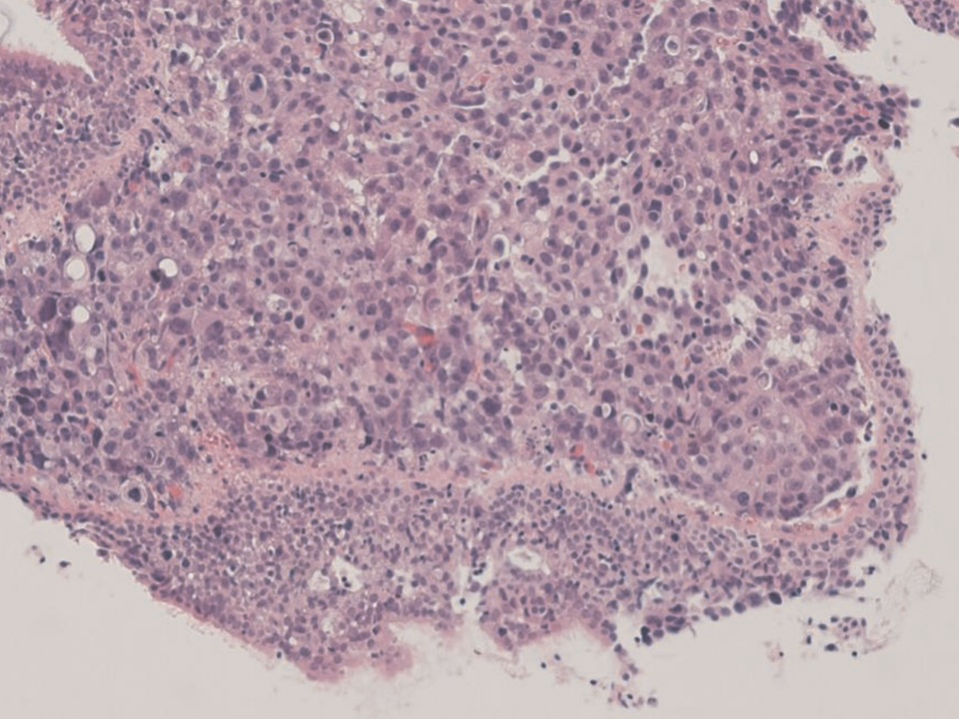
Microscopic images of bronchoscopic biopsy hematoxylin‐eosin staining (magnification × 200) showed large polygonal carcinoma cells similar to small intestine tumor cells.

Fiberoptic bronchoscopic biopsy was performed, and lung cancer confirmed. The lung cancer histology was similar to that of small intestinal cancer. The tumor cells were large, polygonal, with abundant cytoplasm. Some of the tumor cells showed rhabdoid features which was the same as the tumor cells in the small intestine (Fig 4). The tumor cells lacked cytological and architectural features of neuroendocrine tumor cells, adenocarcinoma or squamous cell carcinoma. Immunohistochemically, the tumor cells did not express TTF‐1, p40 or any neuroendocrine markers; however, they expressed cytokeratin and the cancer was subsequently diagnosed as NSCLC, not otherwise specified (NOS). Because primary small intestinal carcinoma, especially extraduodenal carcinoma, is extremely rare and both tumors showed the same histological and immunohistochemical features, we concluded that the small intestinal tumor was lung metastasis.

As this study was a clinical case report, no ethical committee approval was required, which was in compliance with the institutional and national policies concerning research approvals. The patient was informed that clinical details and images concerning the case would be submitted for publication, and provided his consent.

## Discussion

Metastasis from primary lung cancer to the gastrointestinal tract is relatively rare and has been reported to be 0.2%–1.7%,^3^, ^4^, ^5^ but autopsy data show that the rate of metastasis to the gastrointestinal tract is 4.7%–14% higher than that clinically reported.^6^, ^10^ This difference suggests that even if lung cancer metastasizes to the gastrointestinal tract, the associated symptoms and complications are relatively rare.

Metastasis to the small bowel is always accompanied by metastasis to other organs, suggesting that small bowel metastasis only occurs in the advanced stages of lung cancer.^7^ Most patients with small bowel metastasis of lung cancer do not show specific symptoms. Therefore, small bowel metastasis is often diagnosed only after disease progression, and in most cases, emergency surgery is required owing to life‐threatening complications such as obstruction, perforation, and bleeding.^8^, ^9^ In a review of 58 cases of small bowel metastasis of lung cancer, the majority of patients were male, aged 36–78 years, and the complications of small bowel metastasis were perforation (59%), obstruction (29%), and bleeding (10%).^11^

As in the case reported here, sometimes the differential diagnosis between primary and metastatic carcinoma is not easy, and more than 50% of small intestinal cancer is metastatic tumor from other sites.^12^ Usually, advanced peritoneal carcinomatosis of adjacent organs such as stomach, pancreas and colon are common primary sites. However, hematogenous metastasis from melanoma, lung and breast can also occur. These primary and metastatic tumors are therefore major differential diagnoses of intestinal malignancy. If there are other tumors in different organs or a history of other malignancy, the small intestinal tumor is more likely to be metastasis. Therefore, in addition to the histological study, extensive imaging such as PET‐CT is necessary to determine a candidate for primary sites. Comparison of histological and immunohistochemical features of each obtained from multiple sites may also be helpful.

The optimal treatment and management strategy of patients with primary lung cancer presenting with symptomatic gastrointestinal metastasis remains controversial. Some studies have recommended conservative treatment owing to poor outcomes, and others have recommended aggressive surgery as it offers effective palliation.^13^ This suggests that an individualized treatment approach should be considered.

Thus, primary lung cancer metastases to the gastrointestinal tract are rare with no associated symptoms; and therefore, the diagnosis is often delayed. Small bowel metastasis is often accompanied by severe complications. Surgical treatment may be considered as palliative treatment depending on the patient's condition. Physicians should be aware of gastrointestinal metastasis in advanced lung cancer in clinical practice.

## Disclosure

The authors report that there are no conflicts of interest.
